# A green and facile synthesis of an industrially important quaternary heterocyclic intermediates for baricitinib

**DOI:** 10.1186/s13065-019-0639-y

**Published:** 2019-10-31

**Authors:** Xin Cui, Junming Du, Zongqing Jia, Xilong Wang, Haiyong Jia

**Affiliations:** 1 School of Pharmacy, Weifang Medical University, Weifang, 261053 Shandong People’s Republic of China; 2 Shandong Peninsula Engineering Research Center of Comprehensive Brine Utilization, Weifang University of Science and Technology, Weifang, 262700 Shandong People’s Republic of China; 3 Shanghai Daozhen Pharmaceutical Technology Co., LTD, Shanhai, 201400 People’s Republic of China

**Keywords:** Baricitinib, JAK1/JAK2 inhibitor, Green synthesis, Microchannel reactor

## Abstract

**Background:**

Baricitinib, with a 2-(1-(ethylsulfonyl)azetidin-3-yl)acetonitrile moiety at *N*-2 position of the pyrazol skeleton, is an oral and selective reversible inhibitor of the JAK1 and JAK2 and displays potent anti-inflammatory activity. Several research-scale synthetic methods have been reported for the preparation of key quaternary heterocyclic intermediates of baricitinib. However, they were all associated with several drawbacks, such as the expensive materials, usage of pollutional reagents, and poor yields.

**Results:**

In this manuscript, we established a green and cost-effective synthesis of 2-(1-(ethylsulfonyl)azetidin-3-ylidene)acetonitrile and tert-butyl 3-(cyanomethylene)azetidine-1-carboxylate for further scale-up production of baricitinib. This synthetic method employs commercially available and low-cost starting material benzylamine and an industry-oriented reaction of green oxidation reaction in microchannel reactor to yield important quaternary heterocyclic intermediates.

**Conclusion:**

Generally, this procedure is reasonable, green and suitable for industrial production.

## Background

**Fig. 1:**
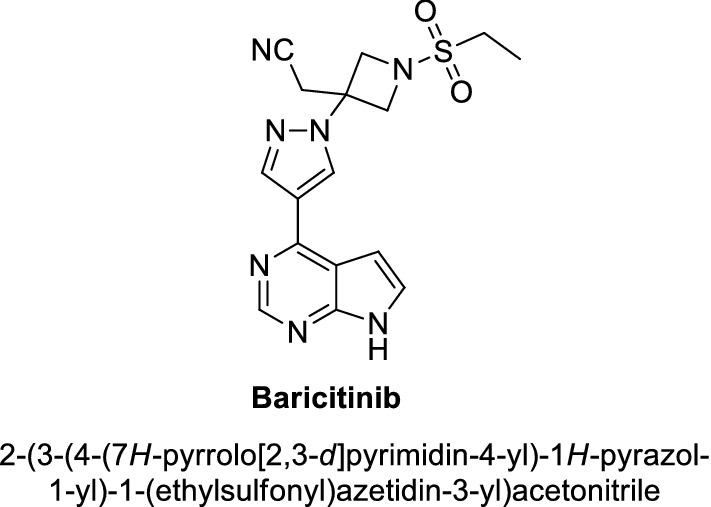
Structure of lesinurad baricitinib

Baricitinib, with a 2-(1-(ethylsulfonyl)azetidin-3-yl)acetonitrile moiety at the N-2 position of the pyrazol skeleton (Fig. 1), is an oral and selective reversible inhibitor of the JAK1 and JAK2 and displays potent anti-inflammatory activity [1, 2]. Besides, baricitinib has also been approved by the European Union in March 2017 and Japan in July 2017 for the treatment of moderate to severe rheumatoid arthritis for inhibiting the intracellular signaling of many inflammatory cytokines such as IL-6 and IL-23 [3–5] and for the patients with rheumatoid arthritis and poor response to the current standard treatment [2], respectively. For the above, the synthetic method of baricitinib has drew great attentions and been thoroughly investigated [1, 2] in recent years.

**Scheme 1:**
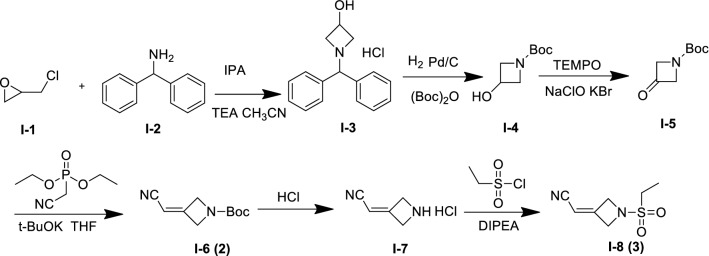
Synthesis of intermediate 2 and 3 using 2-(chloromethyl)oxirane (I-1) and diphenylmethanamine (I-2) as starting material

**Scheme 2:**
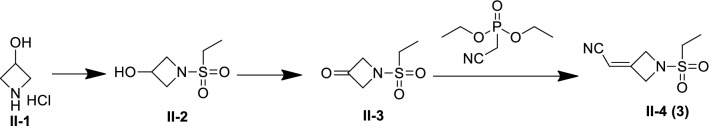
Synthesis of intermediate 3 with II-1 as starting material

**Scheme 3:**
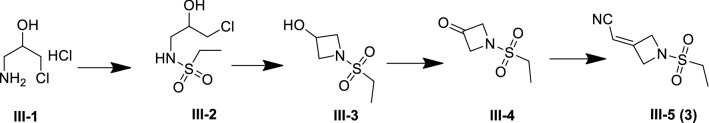
Synthesis of intermediate 3 with III-1 as starting material

**Scheme 4:**
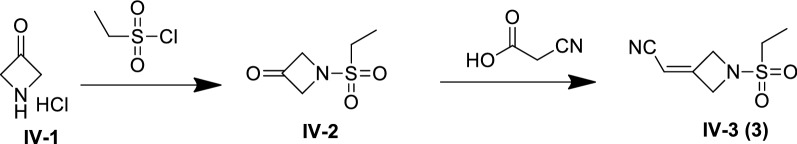
Synthesis of intermediate 3 with IV-1 as starting material

Almost all the synthetic methods (WO2009114512A1, CN201510880931.X, CN201610080433.1, WO2016088094A1, WO2016125080A2, WO2016205487A1, CN201610903498.1, WO2017109524A1, CN201710181322.4, CN201710165830.3) reported for the preparation of baricitinib employed important intermediates 2-(1-(ethylsulfonyl)azetidin-3-ylidene)acetonitrile(**2**) and tert-butyl 3-(cyanomethylene)azetidine-1-carboxylate(**3**), for which the development of a green and facile synthetic method for intermediates **2** and **3** has a strong demand. However, several reported research-scale synthetic methods for the preparation of intermediates **2** and **3** (Schemes 1, 2, 3 and 4) were associated with several drawbacks, such as the expensive materials, usage of pollutional reagents, poor yields, and so on. In this paper, we describe a green and facile synthesis of key quaternary heterocyclic intermediates (**2** and **3)**.

### Synthesis process of key quaternary heterocyclic intermediates (**2** and **3**)

The main medicinal chemistry routes of quaternary heterocyclic intermediates (2 and 3) are outlined in Schemes 1, 2, 3 and 4. (1) In Scheme 1, compounds 2-(chloromethyl)oxirane (**I-1**) and diphenylmethanamine **(I-2**) were used as the starting material (WO2009114512A1). Intermediate 2 was obtained through reduction reaction, boc-protecting reaction, oxidizing reaction, and wittig reaction, which was then employed to afford intermediate 3 by deprotect and hinsber reactions [6–8]. (2) In Scheme 2, compound azetidin-3-ol hydrochloride (II-1) was used as start material, which was employed to afford intermediate 3 through hinsber reaction, oxidizing reaction, and wittig reaction (WO2016205487A1) [9]. Besides, another patent reported that the start material 1-amino-3-chloropropan-2-ol hydrochloride (III-1) was first reacted with ethanesulfonyl chloride to afford compound N-(3-chloro-2-hydroxypropyl)ethanesulfonamide (III-2), which was then converted to the same intermediate 1-(ethylsulfonyl)azetidin-3-ol (III-3, II-2) after cyclization. Key intermediate 3 was obtained by the same method as that of Scheme 2 **(**Scheme 3, CN201710165830.3). (3) In Scheme 4, compound azetidin-3-one hydrochloride (IV-1) was used as raw start material, which was converted to intermediate 3 through hinsber reaction and aldol condensation reaction **(**CN201610903498.1).

However, the above synthetic methods have several defects. In Scheme 1, the yield of the first step is just only 43.4%, and the byproduct diphenylmethane in the second step is difficult to remove. Besides, in the third step, it will produce a large amount of mixed salt wastewater, which will bring great pressure to environmental protection and non-suitable for industrial production. In Schemes 2, 3, 4, the start materials are too expensive, which are also non-suitable for industrial production. Therefore, these drawbacks prompted us to consider some alternative approaches to synthesize the intermediates 2 and 3. Herein, we presented our efforts for the development of a green and facile synthetic route with increased overall yield and suitable for industrial production, which were summarized in this manuscript.

## Results and discussion

**Scheme 5:**
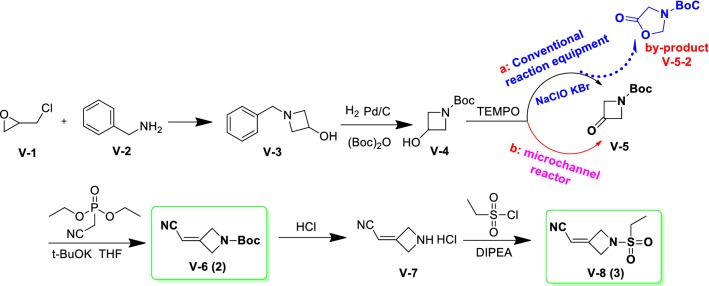
A green and facile synthesis of intermediate 3

A novel and green synthetic procedure was successfully demonstrated to generate laboratory-scale key quaternary heterocyclic intermediate 3 in six steps (Scheme 5). The route started with the cheaper and commercially available 2-(chloromethyl)oxirane (**V-1**) and benzylamine (**V-2**), which was converted to 1-benzylazetidin-3-ol(**V-3**). Compound V-3 was then converted via reduction reaction and N-Boc protection to afford compound **V-4**, which was reacted with 2,2,6,6-tetramethylpiperidine-1-oxyl (TEMPO) to obtain intermediate V-5 by two different methods. Then intermediate **V-5** was employed to afforded key intermediates tert-butyl 3-(cyanomethylene)azetidine-1-carboxylate (**V-6**, 2)and 2-(1-(ethylsulfonyl)azetidin-3-ylidene)acetonitrile (**V-8**, 3) successively underwent wittig reaction, deprotection, and hinsber reactions.

In this green and facile synthetic route, we used benzylamine as the starting material instead of unstable reagent benzhydrylamine compared with the synthetic route in Scheme 1, as benzhydrylamine will be partly converted to dibenzophenone. Besides, the starting material benzylamine was much cheaper than benzhydrylamine, which was more suitable for industrial production. Moreover, in the second step, the by-product of deprotected toluene can be more easily removed by rectification process compared to the by-product diphenylmethane in the synthetic route in Scheme 1.

**Fig. 2:**
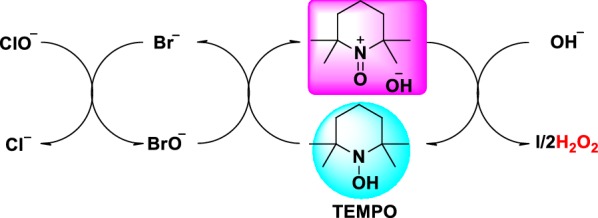
The process of producing peroxide

At first, traditional TEMPO reaction (sodium hypochlorite as an oxidant) in the third step was employed. Alkali with different concentrations were employed to reduce wastewater output and increase the yield. However, the by-product **V-5-2** (tert-butyl 5-oxooxazolidine-3-carboxylate) was always yielded no matter how the reaction conditions were changed (Table 1). The effects of different temperatures on the ratios of product and by-product were shown in Table 1, which suggested that − 10 °C was optimal temperature. Besides, we found that compound V-5 was converted to by-product V-5-2 by peroxidation and rearrangement reaction (Baeyer–Villiger oxidation rearrangement reaction). Peroxide H_2_O_2_ was produced first as the following process (Fig. 2), which urged V-5 to by-product V-5-2 through Baeyer–Villiger oxidation rearrangement reaction.

**Table 1:** Optimization of reaction conditions

Entry	Solvent	Temperature (°C)	Time	V-5/V-5-2 (mol/mol)
1	DCM	5	30 min	76.0/24.0
2	DCM	0	30 min	90.9/9.1
3	DCM	− 5	30 min	95.7/4.3
4	DCM	− 10	30 min	97.6/2.4
5	DCM	− 15	1.5 h	97.1/2.9

Though lots of conditions screened, by-product V-5-2 was just controlled in 5% by traditional TEMPO reaction. To solve this problem, microchannel reactor was used with two methods instead of traditional TEMPO reaction, as it has the advantage of high heat efficiency and mass transfer property.

**Fig. 3:**
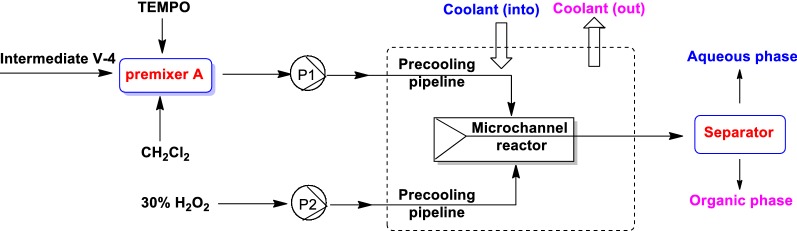
The flow diagram of synthesize intermediate V-5 in method 1

Method 1: TEMPO-H_2_O_2_ system (Fig. 3), shortening residence time of product, inhibited the yield of by-product V-5-2, which reduced salt mixing wastewater and can be directly access to the sewage plant. In this step, the equivalents of V-5, TEMPO and H_2_O_2_ was 1: 0.02: (2–10) and the best temperature was among 0–30 °C.

**Fig. 4:**
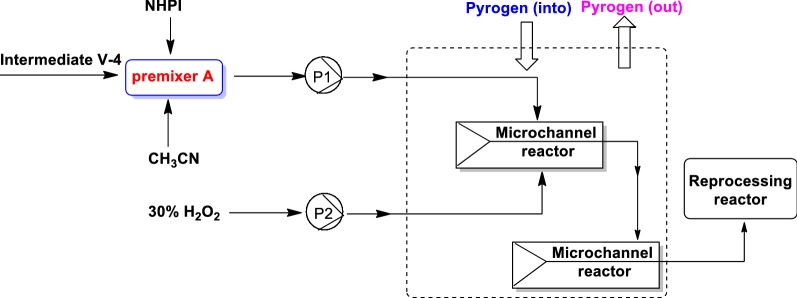
The flow diagram of synthesize intermediate V-5 in method 1

Method 2: Composite catalysts—O_2_ system (Fig. 4), the advanced system, do not produce by-product V-5-2, which fundamentally resolved the mixed salt wastewater. In this method, catalysts and cocatalysts were included in composite catalysts. Catalysts were including cobalt acetate or manganese acetate, and cocatalysts were including *N*-hydroxybenzoyl dimethylimide or 3-chlorobenzoic acid. The equivalents of V-5, catalysts, and cocatalysts was 1: (0.01–0.1): (0.01–0.1) and the proper temperature was among 25–75 °C.

## Conclusions

In conclusion, we provide a green and facile synthesis of an industrially important quaternary heterocyclic intermediate for baricitinib, which proceeds in six steps with multiple advantages. The most significant step of the route is the synthesis of intermediate tert-butyl 3-oxoazetidine-1-carboxylate (**V-5**), and there are many advantages of this method, such as inexpensive starting materials, less by-product, easily work up, and environmental protection. Moreover, the reaction reactant, reaction time, temperature, and solvent of this step were preliminarily investigated. This environmental-friendly, cost-effective and facile process and the optimum conditions for the preparation of quaternary heterocyclic intermediates for baricitinib may form the basis of a future manufacturing route.

## Experimental section

^1^H NMR spectra was obtained on a Bruker AV-400 spectrometer (Bruker BioSpin, Fällanden, Switzerland) in the indicated solvent CDCl_3_. Chemical shifts were expressed in δ units (ppm), using TMS as an internal standard, and *J* values were reported in hertz (Hz). TLC was performed on Silica Gel GF254. Spots were visualized by irradiation with UV light (λ 254 nm). Flash column chromatography was carried out on columns packed with silica gel 60 (200–300 mesh). Solvents were of reagent grade and, if needed, were purified and dried by distillation. Starting materials, solvents, and the key reagents were purchased from commercial suppliers and were used as received without purification.

### General procedure for the synthesis of 1-benzylazetidin-3-ol (V-3) [10–12]

To the solution of benzylamine (30.0 g, 324 mmol) in water (450 mL) 2-(chloromethyl)oxirane (30.0 g, 280 mmol) was slowly added under 0–5 °C. The reaction mixture was stirred at 0–5 °C for 16 h. Upon completion of the reaction, the crude product was isolated by filtration, washed with water (60 mL) and dried in vacuo, which was dissolved in CH_3_CN (485 mL) and was added in portions Na_2_CO_3_ (42.0 g, 396 mmol). The mixture solution was then heated to 80–90 °C and stirred for 16 h under reflux. Upon completion of the reaction by TLC, the residue was concentrated to obtain viscous white solid. To the mixture solution of above viscous white solid in methyl tert-butyl ether (MTBE, 180 mL) were slowly added with oxalic acid (28 g, 311 mmol) in MTBE (140 mL). After the reaction mixture was stirred at room temperature for 3 h, the crude product was isolated by filtration, which was dissolved in ethyl acetate (300 mL) again and washed with 10% Na_2_CO_3_ (50 mL × 3). The organic layer was concentrated under vacuum to give the desired compounds V-3 as a solid (39.6 g, 88.7% yield). ^1^H-NMR (400 MHz, CDCl_3_) δ ppm: 2.40–2.46 (m,1H), 2.96–2.99 (m,2H), 3.60–3.70 (m,4H), 4.40–4.44 (m,1H), 7.21–7.34 (m,5H).

### General procedure for the synthesis of tert-butyl 3-hydroxyazetidine-1-carboxylate (V-4)

To the mixture solution of 1-benzylazetidin-3-ol (V-3) (35.0 g, 214.4 mmol) in THF (350 mL) was added with 5% Pd/C (1.75 g). The reaction mixture was stirred at room temperature overnight under H_2_ atmosphere for 20 h. Upon completion of the reaction, the reaction mixture was filtered by a suction filter and the filtrated was removed under vacuum and giving the desired crude compound tert-butyl 3-hydroxyazetidine-1-carboxylate (V-4). It was dissolved in n-heptane (105 mL) and stirred with 0-5 °C for 2 h under N_2_ atmosphere, which was filtered again and the filter cake was dried to afford pure white solid V-4 (33.8 g, 91% yield). ^1^H NMR (400 MHz, CDCl_3_) δ ppm: 1.40 (s,1H),3.76–3.78 (m,2H), 4.08–4.10 (m,2H), 4.51–4.55 (m,1H).

### General procedure for the synthesis of t tert-butyl 3-oxoazetidine-1-carboxylate (V-5) (traditional TEMPO reaction with oxidant NaClO)

To the solution of tert-butyl 3-hydroxyazetidine-1-carboxylate (V-4, 10.0 g, 57.7 mmol) in CH_2_Cl_2_ (200 mL) 9.1% potassium bromide water solution (15.1 g) and TEMPO (0.18 g, 1.15 mmol) were slowly added under − 15 to 5 °C, which was added the mixture solution of KHCO_3_ (104 g) and NaClO (86 g, 12% water solution) in water (389 mL) and stirred for half an hour. Upon completion of the reaction, the reaction mixture was quenched by 15% sodium thiosulfate aqueous solution (100 mL), extracted with ethyl acetate, washed with water, and then the solvent was removed under vacuum. The residue was dissolved in ethyl acetate again, which was added slowly 5 mL *n*-heptane and 0.1 g seed crystal under 10–15 °C with stirred for 20 min. And then another 5 mL *n*-heptane was added under − 5–0 °C and stirred for 20 min. The mixture was filtered and the filter cake was dried to afford desired compound V-5 with little by product V-5-2. Compound V-5 ^1^H NMR (400 MHz, CDCl_3_) δ ppm: 1.45(s,9H), 4.65(s,4H); Compound V-5-2 ^1^H NMR (400 MHz, CDCl_3_) δ ppm: 1.46(s,9H), 3.97(s,2H), 5.32(s,2H).

### General procedure for the synthesis of t tert-butyl 3-oxoazetidine-1-carboxylate (V-5) (the microchannel reactor with TEMPO-H_2_O_2_ system)

Intermediate tert-butyl 3-hydroxyazetidine-1-carboxylate (V-4, 10.0 g, 57.7 mmol), TEMPO (0.18 g, 1.15 mmol) and CH_2_Cl_2_ (120 mL) were added in premixed reactor A, which was derived to the micro-channel reactor with the speed of 6.5 g/min. Meanwhile, 30% H_2_O_2_ solution was pumped into the micro-channel reactor at a speed of 4.5 g/min and the stay time was 30 s. Upon completion of the reaction, the mixture solution was pumped into oil–water separator for 20 min. The organic phase was washed by water (20 mL), concentrated under vacuum to give the residue, which was dissolved in 15 mL *n*-heptane under 30 °C. Then 0.1 g seed crystal was added under 10–15 °C and stirred for 20 min, which was stirred for another 20 min under − 5–0 °C. The mixture was filtered and the filter cake was dried to afford desired compound V-5 (9.1 g, 92.1% yield) without by-product V-5-2. HPLC: 99.07%.

### General procedure for the synthesis of t tert-butyl 3-oxoazetidine-1-carboxylate (V-5) (the microchannel reactor with composite catalyst-O_2_ system)

Intermediate tert-butyl 3-hydroxyazetidine-1-carboxylate (V-4, 5.0 g, 28.8 mmol), *N*-hydroxyphthalimide (0.94 g, 5.76 mmol) and CH_3_CN (50 mL) were added in premixed reactor A, which was derived to the micro-channel reactor with the speed of 1 mL/min. Meanwhile, the solution of cobalt acetate (0.14 g cobalt acetate in 25 mL acetic acid) was pumped into the micro-channel reactor at a speed of 4.5 g/min and the stay time was 90 s. Upon completion of the reaction, the mixture solution was pumped into treatment reactor for 55 min. The reaction solution was concentrated and 50 mL CH_2_Cl_2_ was added, which was washed by 20 mL water and 20 mL salt solution, dried to afford white crude solid. The above solid was dissolved in 2.5 mL ethyl acetate under 15 °C, which was added slowly 5 mL *n*-heptane and 0.1 g seed crystal with stirred for 20 min under − 5–0 °C. The mixture was filtered and the filter cake was dried to afford desired compound V-5 (4.3 g, 87% yield) without by-product V-5-2. HPLC: 99%.

### General procedure for the synthesis of tert-butyl 3-(cyanomethylene)azetidine-1-carboxylate (V-6)

To the solution of diethyl (cyanomethyl)phosphonate (24.8 g, 140 mmol) in THF (300 mL) potassium tert-butoxide solution of THF (128.5 mL, 1 mol/L) was slowly added under H_2_ atmosphere, which was stirred under − 5 °C for 3 h. Then the intermediate tert-butyl 3-oxoazetidine-1-carboxylate (V-5, 20.0 g, 116.8 mmol), dissolved in 67 mL THF, was added and continue stirred for another 2 h under − 5 °C. The mixture solution was warmed to room temperature and continue reacted for 16 h. Upon completion of the reaction, an aqueous solution of sodium chloride (12.5%, 300 mL) was added, which was extracted by ethyl acetate (100 mL × 3). And then the organic phrase was washed by saturated salt solution (200 mL), concentrated under vacuum to give the desired compounds V-6 as a white solid (20.7 g, 91% yield). ^1^H NMR (400 MHz, CDCl_3_) δ ppm: 1.44 (s, 9H), 4.60 (s, 2H), 4.69 (s, 2H), 5.37 (s, 1H).

### General procedure for the synthesis of 2-(1-(ethylsulfonyl)azetidin-3-ylidene)acetonitrile (V-7)

To the solution of tert-butyl 3-(cyanomethylene)azetidine-1-carboxylate (V-6, 36.0 g, 185 mmol) in CH_3_CN (252 mL) hydrochloric acid (252 mL, 3 mol/L) was added and stirred under room temperature for 16 h. After completion of the reaction, the mixture solution was concentrated under vacuum and dissolved in 144 mL CH_3_CN, which was stirred for 2 h under 30 °C. And then the solution was cooled to 5 °C and stirred for another 2 h. The mixture was filtered and the filter cake was dissolved in 432 mL CH_3_CN. Diisopropylethylamine (97.1 mL) and ethanesulfonyl chloride (26.3 mL) were added under 15 °C. The reaction mixture was stirred for 12 h under 20 °C. Upon completion of the reaction, the mixture solution was concentrated under vacuum, dissolved in 360 mL CH_2_Cl_2_, extracted by 180 mL 12.5% aqueous solution of NaCl, concentrated under vacuum again to afford crud compound 2-(1-(ethylsulfonyl)azetidin-3-ylidene)acetonitrile (V-7). The crud compound V-7 was dissolved in 36 mL ethyl acetate and warmed to 50 °C. *N*-Heptane (48 mL) was added and cooled to 30 °C. Then 0.2 g seed crystal was added and stirred for 20 min, another n-heptane (48 mL) was added, stirred for 50 min under − 5 to 0 °C. The mixture was filtered and the filter cake was dried to afford pure compound V-7 (30.5 g, 88.4% yield). ^1^H NMR (400 MHz, CDCl_3_) δ ppm: 1.37 (t, *J *= 4.8, 3H), 3.03 (q, *J *= 4.8, 2H), 4.83 (s, 2H), 4.76 (d, *J *= 1.2, 2H), 5.43 (d, *J *= 1.2, 1H).

## Supplementary information

**Additional file 1.** Copies of NMR and MS spectra.

## Data Availability

All data generated or analysed during this study are included in this published article and its Additional file 1.

## Abbreviations

JAK1: Janus kinase 1
JAK2: Janus kinase 2
TEMPO: 2,2,6,6-tetramethylpiperidine 1-oxyl
