# Supplementary figures and images for: A green and facile synthesis of an industrially important quaternary heterocyclic intermediates for baricitinib
Source: BMC Chem. 2019 Oct 31;13(1):123. doi: 10.1186/s13065-019-0639-y (PMC6824028; doi:10.1186/s13065-019-0639-y)

Compound V-3 1HNMR


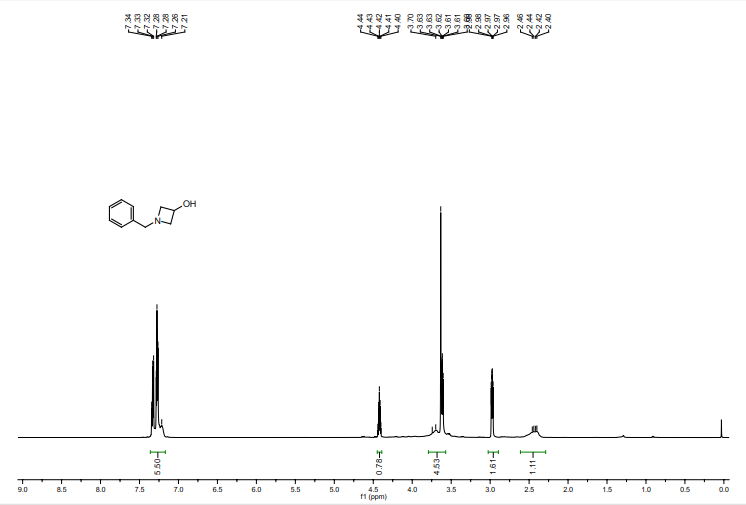


Compound V-4 1HNMR


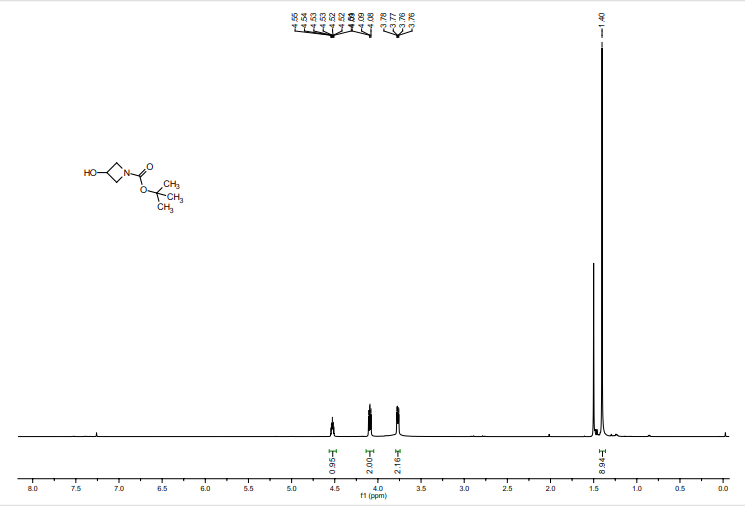


Compound V-5 1HNMR


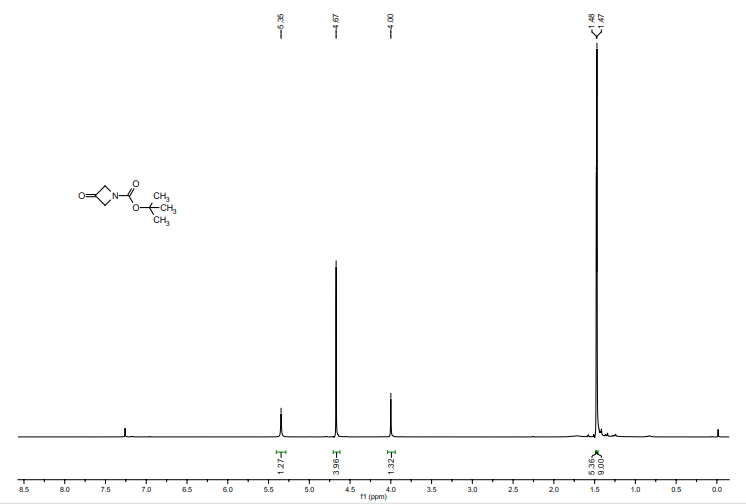


Compound V-5 MS


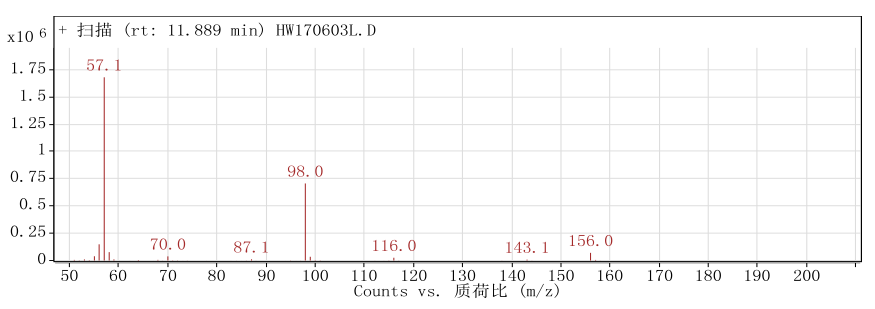


Compound 7 1HNMR


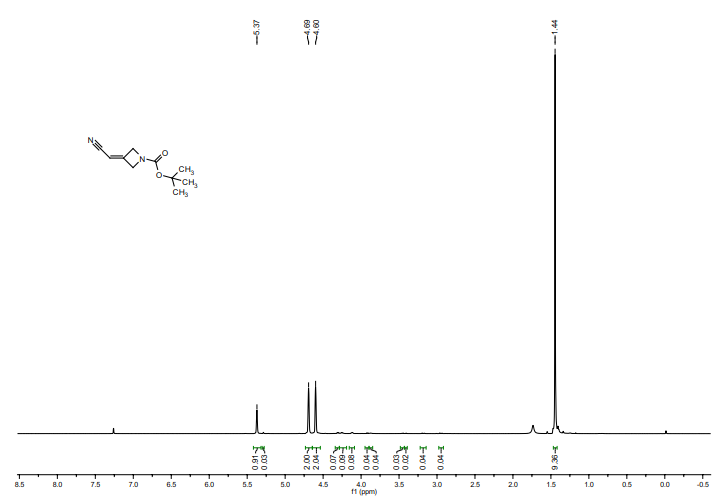


Compound 8 1HNMR


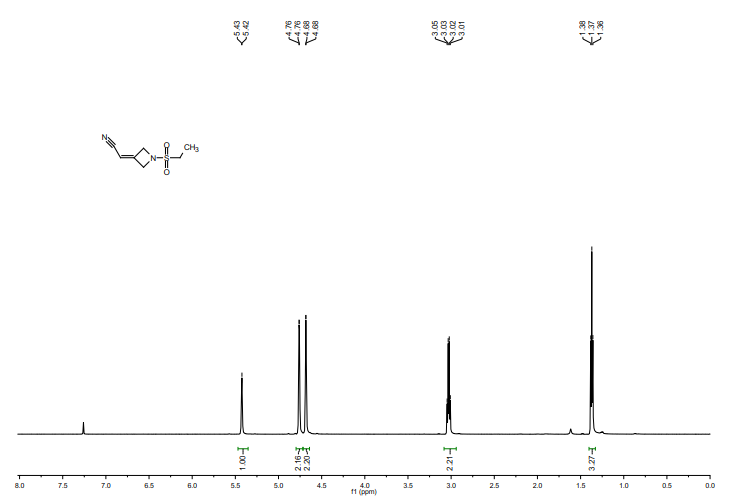

Supplement: Supplementary file 1 — Additional file 1. Copies of NMR and MS spectra. [file 13065_2019_639_MOESM1_ESM.docx]
